# Clinical and cost effectiveness of computer treatment for aphasia post stroke (Big CACTUS): study protocol for a randomised controlled trial

**DOI:** 10.1186/s13063-014-0527-7

**Published:** 2015-01-27

**Authors:** Rebecca Palmer, Cindy Cooper, Pam Enderby, Marian Brady, Steven Julious, Audrey Bowen, Nicholas Latimer

**Affiliations:** School of Health and Related Research, University of Sheffield, 107 Innovation Centre, 217 Portobello, Sheffield, S1 4DP England; Nursing, Midwifery and Allied Health Professions Research Unit, Glasgow Caledonian University, Cowcaddens Road, Glasgow, G40BA England; Medical Statistics Group, School of Health and Related Research, University of Sheffield, Regent Court, Regent Street, Sheffield, S1 4DA England; Reader, School of Psychological Sciences, University of Manchester (MAHSC), Joint theme lead - Patient-Centred Care, NIHR CLAHRC Greater Manchester, Centre for Stroke and Vascular Research, CSB, Salford Royal NHS Foundation Trust, Stott Lane, Salford, M6 8HD England; Senior Research Fellow in Health Economics, Health Economics and Decision Science, ScHARR, University of Sheffield, Regent Court, 30 Regent Street, Sheffield, S1 4DA England

**Keywords:** Speech and language therapy, Aphasia, Computerised intervention, Self-management, Long term rehabilitation, RCT design, Stroke

## Abstract

**Background:**

Aphasia affects the ability to speak, comprehend spoken language, read and write. One third of stroke survivors experience aphasia. Evidence suggests that aphasia can continue to improve after the first few months with intensive speech and language therapy, which is frequently beyond what resources allow. The development of computer software for language practice provides an opportunity for self-managed therapy. This pragmatic randomised controlled trial will investigate the clinical and cost effectiveness of a computerised approach to long-term aphasia therapy post stroke.

**Methods/Design:**

A total of 285 adults with aphasia at least four months post stroke will be randomly allocated to either usual care, computerised intervention in addition to usual care or attention and activity control in addition to usual care. Those in the intervention group will receive six months of self-managed word finding practice on their home computer with monthly face-to-face support from a volunteer/assistant. Those in the attention control group will receive puzzle activities, supplemented by monthly telephone calls.

Study delivery will be coordinated by 20 speech and language therapy departments across the United Kingdom. Outcome measures will be made at baseline, six, nine and 12 months after randomisation by blinded speech and language therapist assessors. Primary outcomes are the change in number of words (of personal relevance) named correctly at six months and improvement in functional conversation. Primary outcomes will be analysed using a Hochberg testing procedure. Significance will be declared if differences in both word retrieval and functional conversation at six months are significant at the 5% level, or if either comparison is significant at 2.5%. A cost utility analysis will be undertaken from the NHS and personal social service perspective. Differences between costs and quality-adjusted life years in the three groups will be described and the incremental cost effectiveness ratio will be calculated. Treatment fidelity will be monitored.

**Discussion:**

This is the first fully powered trial of the clinical and cost effectiveness of computerised aphasia therapy. Specific challenges in designing the protocol are considered.

**Trial registration:**

Registered with Current Controlled Trials ISRCTN68798818 on 18 February 2014.

## Background

Stroke is the largest cause of disability in the United Kingdom with communication impairment affecting one third of survivors [1]. Aphasia is the most common communication impairment acquired post stroke. It is a disorder of language which may affect understanding, expression, reading and writing. Speech and language therapy (SLT) is often received regularly initially but rarely continues after the first few months. Medical instability, fatigue and confusion may reduce full engagement with language therapy in the early weeks post stroke, reducing the opportunity for people to participate in treatment. There is evidence that people can continue to improve their language skills for several years [2]. As the consequences of aphasia remain a problem long term, investigation of interventions to reduce this health burden in the chronic stages post stroke is crucial. The National Stroke Strategy [1] recommends people receive rehabilitation for as long as they benefit from it. Treatment of aphasia that persists beyond the first few months post stroke is often not available through NHS services in the United Kingdom as ongoing therapy is costly through face-to-face SLT and places greater demands on limited resources.

Meta-analysis in a Cochrane review [2] of SLT for aphasia following stroke suggests some effectiveness of aphasia therapy [2]. Adequately powered randomized controlled trials (RCTs) in this field are rare except for recent studies of SLT intervention in the first few weeks post stroke. Laska *et al*. [3] randomised 123 patients with aphasia to receive 45 minutes of SLT a day for 21 days, starting within two days of stroke onset, or no SLT intervention. Severity of the aphasia was not reduced. A recently completed study, ACT NoW [4], randomised 170 people in hospital post stroke to SLT intervention or attention control (informal conversation with paid visitors) for up to four months. No significant differences between groups were shown and the authors suggested that the intervention may have been provided too early in the stroke pathway. As aphasia persists for many stroke survivors, therapy in the longer term also warrants investigation using adequately powered RCTs. Although rapid spontaneous recovery may occur in the first few months, there is preliminary evidence to suggest targeted and intensive SLT treatments can promote further improvement in the latter months [5-7].

Targeted therapies with good preliminary evidence to date include constraint induced aphasia therapy (CIAT); use of language in games to make, reject or clarify requests for targeted items for 30 hours over 2 weeks [5,8,9]. A systematic review of 10 studies conducted over the decade concluded that evidence for this technique is favourable [10]. Model oriented aphasia therapy (MOAT), which tailors treatment according to a patient’s individual symptoms, was found to be comparable to CIAT when delivered with similar intensity [11]. Raymer *et al*. found personal relevance or ‘salience’ of the language material being practiced to be important when targeting therapy [6]. While the optimum intensity remains unclear, it is generally acknowledged that for stroke rehabilitation regular, repetitive therapy practice is a factor in treatment success.

The resources required to achieve intensive therapy in the long term is prohibitive in the current financial climate and lower cost options for the support of repetitive intensive practice are needed. Non-speech and language therapy professionals have been employed successfully to support therapy activity [4,12,13].

Computer therapy, developed for the treatment of aphasia, has been reported to be useful in the provision of targeted language practice and provides opportunities for independent home practice as part of a self-management approach to maximise practice intensity, improving outcomes for reading, spelling and expressive language [13-17]. The Department of Health report, ‘*Our Health, Our Care, Our Say*’ [18], recommends self-management for long term conditions supported through technological innovation [18]. However, to date, studies of self-managed computer therapy for aphasia have been limited to descriptive case series with only three reported RCTs; two for treatment of reading disorders and one for treatment of word finding [13,14,19]. Although these studies were not fully powered, they indicate potential effectiveness of computer therapy. Such computer-based services for long term management of aphasia therapy could provide a low cost therapy option which is acceptable to patients and families. However, the actual cost effectiveness has not been definitively tested.

The StepbyStep© computerised approach to long term aphasia therapy combines current evidence underpinning language therapy with practical considerations of treatment delivery. Skills of a qualified speech and language therapist are used to select individually targeted therapy exercises, computer software is provided for regular self-managed practice of therapy exercises and volunteers or assistant SLTs support language practice and computer use [20]. A pilot study evaluating this approach was carried out with 34 people with persistent aphasia. They were randomly assigned to using computer software designed for treating aphasia, or usual long term care (most frequently this was social support). On average people with aphasia practiced their speech exercises on the computer independently for 25 hours over five months. The therapy showed statistically significant improvement in the ability to use spoken words when compared to usual care (*P* = 0.014). The results indicated that self-managed computer therapy supported by volunteers (a total of four hours on average) could help people with aphasia to continue to practise, improving their vocabulary and confidence talking [13]. Patients and carers found it an acceptable alternative to face-to-face therapy [21]. Self-managed computer therapy could therefore improve the quality of life of people with persistent aphasia at relatively low cost, and exploratory economic analysis has suggested considerable potential for the intervention to prove cost effective [13,22].

The aim of this study is to provide definitive evidence of the clinical and cost effectiveness of targeted, intensive speech and language impairment-based therapy intervention for word finding delivered through self-managed computer exercise for persisting post-stroke aphasia. This builds on the pilot RCT which explored possible effects, and informed measures, feasibility, recruitment rates, adherence, cost effectiveness analysis and a power calculation. The current study was commissioned by the United Kingdom National Institute for Health Research, having considered the limitations in the current evidence base.

### Objectives

The primary objectives of the study are:

1. To establish whether self-managed computerised speech and language therapy for aphasia related word finding problems after stroke increases the ability to use vocabulary of personal importance (impairment).
2. To establish whether self-managed computerised speech and language therapy for word finding problems after stroke improves functional communication ability in conversation (activity).
3. To investigate whether patients receiving self-managed computerised speech and language therapy perceive greater changes in social participation and quality of life (participation).
4. To establish whether self-managed computerised speech and language therapy is cost effective for persistent aphasia post stroke.
5. To identify whether any effects of the intervention are evident 12 months after therapy has begun.

Secondary objectives include investigating the generalisation of treatment to retrieval of untreated words (impairment); the use of treated words in conversation; the carer perception of communication effectiveness (participation) and identification of any possible adverse events. Carers’ own quality of life will be measured. Fidelity to treatment will also be monitored.

## Methods/Design

### Design

The study will use a pragmatic, parallel group randomised controlled adjunct trial design. The intervention under test or attention control will be delivered in addition to usual care. Outcomes will be compared for people who are four months or more post stroke, with persistent aphasia who are randomly allocated to either: usual care, self-managed computerised speech and language therapy in addition to usual care or attention control in addition to usual care.

**Figure 1:**
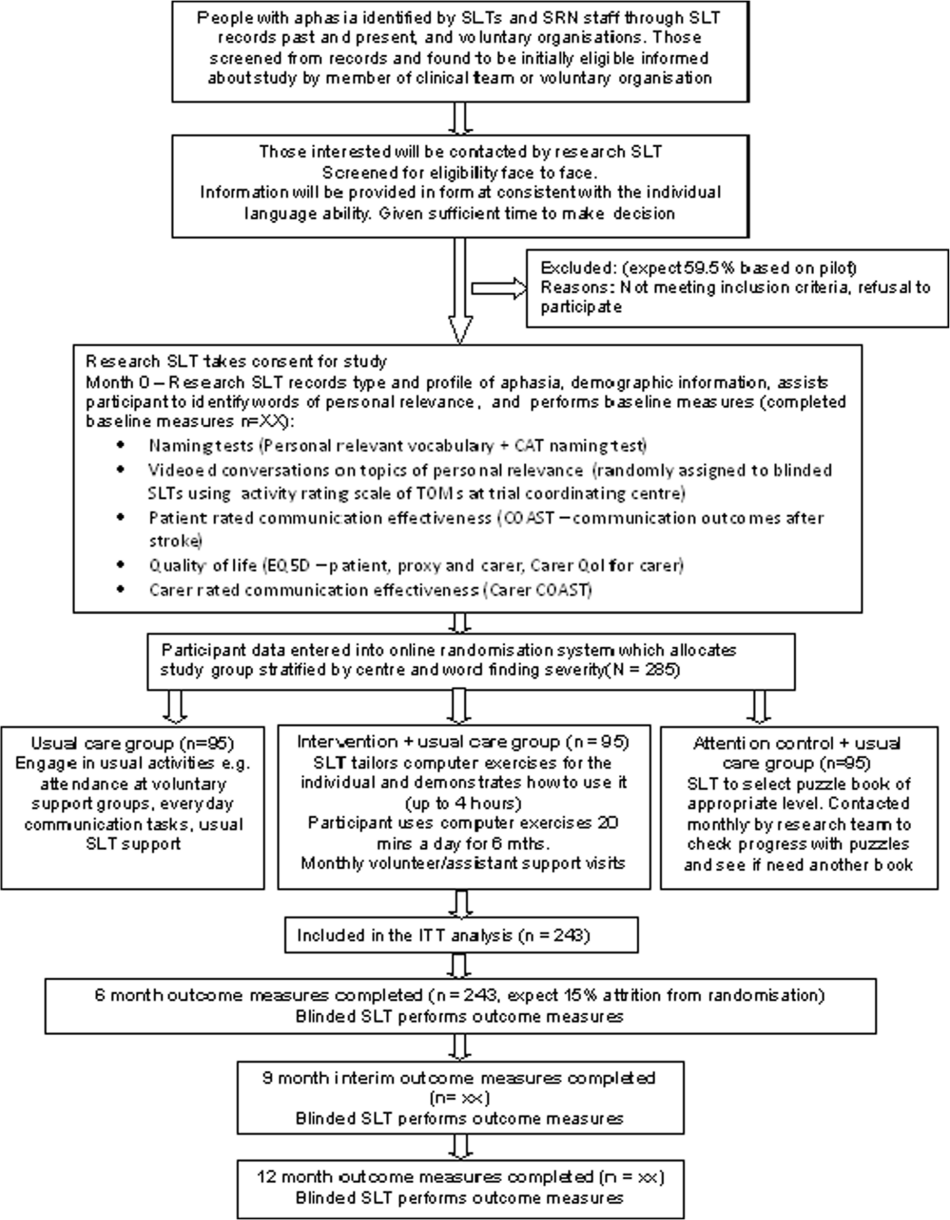
**Progression of participants through the trial (CONSORT diagram).** SLT = speech and language therapist; ITT = intention to treat.

Each participant will be in the trial for 12 months. Participants will be identified and recruited over an 18-month period in total, and 15 months at each site. Each participant will receive their intervention for six months, with follow-up at six, nine and 12 months. The study has an internal pilot phase with criteria for progression to completion of the full RCT. There are no formal statistical criteria for stopping the trial early. Decisions to stop the trial early on grounds of safety or futility will be made by independent data management and ethics committee members. The protocol conforms to the Consolidated Standards of Reporting Trials (CONSORT) guidelines for non-pharmacological studies (Figure 1) [23].

### Ethics approval

The study protocol was approved by Leeds West research ethics committee (reference number: 13/YH/0377). Additional approval was granted for Scotland by the Scotland A research ethics committee (reference number: 14/SS/0023). The University of Sheffield is the sponsor. The trial is registered with the International Standard Randomised Controlled Trials database ISRCTN (reference number: 68798818) and is commissioned by the National Institute for Health Research.

### Participants

A total of 285 participants who have a diagnosis of aphasia as a consequence of a stroke will be recruited from approximately 20 speech and language therapy departments across the United Kingdom. The study will also be advertised at voluntary groups and using posters in libraries and GP surgeries in each locality so that potential participants can self-present to the local research team.

### Inclusion criteria

Participants will be included if they meet the following criteria:

1. Aged 18 or over;
2. Diagnosis of stroke(s);
3. Onset of stroke at least four months prior to randomisation;
4. Diagnosis of aphasia, subsequent to stroke, as confirmed by a trained speech and language therapist;
5. Ability to retrieve 10 to 90% of words on the Comprehensive Aphasia Test Naming Objects subtest (score of 5 to 43 out of 48);
6. Ability to perform a simple matching task in StepByStep with at least 50% accuracy (to confirm sufficient vision and cognitive ability) and
7. Ability to repeat at least 50% of words in a simple word repetition task in the StepByStep© program.

### Exclusion criteria

Participants will be excluded from the study if they meet any of the following criteria:

1. They have another pre-morbid speech and language disorder caused by a neurological deficit other than stroke (a formal diagnosis can be reported by the participant or relatives and confirmed by the recruiting speech and language therapist).
2. They require treatment for a language other than English (as the software is in English).
3. They are currently using the StepbyStep© computer programme or other computer speech therapy aimed at word retrieval and/or naming.

### Procedures

#### Identification

Potential participants will be contacted by the research speech and language therapist in each project centre and provided with information on the project. They will be contacted within a fortnight to establish whether they are interested in knowing more about the study. If they are interested, the research speech and language therapist will make an appointment to visit them at home. The number of those contacted but who cannot be followed up on or who are not interested in learning more and the reason for this will be recorded.

#### Screening for eligibility

Eligibility will be established on the first visit. The speech and language therapist will request verbal consent to carry out the Naming Objects subtest of the Comprehensive Aphasia Test [24]. This test is used in routine practice and will establish the severity of the word finding deficit. If the word finding score is less than 10%, or greater than 90%, an explanation will be given that this type of computer therapy is not suitable for them. If the potential participant has eligible word finding scores, the research speech and language therapist will ask them to try a simple repetition task to confirm their repetition ability, followed by a matching task on the computer to confirm ability to see the screen and perform simple computer related actions.

#### Recruitment

The level of support required to enable a person with aphasia to provide informed consent is dependent upon the severity and profile of the aphasia. In order to provide information in a format consistent with each individual's language ability, a consent support tool (CST) will be used [25]. The research speech and language therapist at each site will request verbal consent from the potential participant to carry out part A of the CST. The result will indicate the style of information they are most likely to understand in part B. Participants will be given sufficient time to consider their participation before informed consent is taken by a research speech and language therapist. Participants providing their own informed consent will be provided with an aphasia-friendly consent form. If potential participants with severe aphasia indicate an interest, a relative (in Scotland this will be the person’s legal representative or nearest relative) will be asked to read an information sheet detailing their responsibility, and will be asked to sign a carer declaration on behalf of their relative with aphasia (in Scotland they will be asked to sign a consent form). For those participants with a carer, the carer will be asked if they are willing to complete some outcome measures related to their own quality of life and perception of their relative’s communication ability. They will be provided with the carer information sheet detailing their potential involvement and will be asked to sign a separate consent form. Informed consent will be obtained from each participant where they are able to give it, or consent or carer declaration will be obtained by the carer or legal representative where the participant is interested in participating, but is unable to provide their own fully informed consent.

### Baseline assessment

Initial assessment will be performed by the local research speech and language therapist once informed consent has been given. This will include collection of the following demographic data: aphasia type, age, gender, time post-onset of stroke and type and location of stroke (if known). Baseline information will also include results of the standardised naming test - Naming Objects subtest of the Comprehensive Aphasia Test (performed as part of eligibility testing during screening) and the Comprehension of Spoken Sentences subtest of the CAT to provide information on the comprehension ability of the participants [24]. Baseline measures relating to the study outcomes are summarised in Table 1.

**Table 1:** **Summary of measures**

**Outcome**	**Measure**	**Method of collection**
Change in word finding ability	Naming of 100 personally relevant words	Taken at baseline by blinded SLT recruiting participant 6, 9 and 12 months by separate blinded SLT.
Change in functional communication	10-minute videoed conversations structured around topics of personal interest. Activity scale of TOMS used to measure conversational ability	Conversations at baseline by blinded SLT. Separate blinded SLT follows same topic guide at 6, 9 and 12 months. Videos randomised and rated centrally by blinded assessors.
Change in patient perception of communication and quality of life	COAST self-reported questionnaire.	Administered by blinded SLT at baseline. Separate blinded SLT at 6, 9, and 12 months.
Generalisation to untreated words	Naming Objects subtest from Comprehensive Aphasia Test	As above
QALYs for cost effectiveness	EQ-5D for patient and carer (accessible and by proxy)	As above
Carer quality of life	Carer COAST and CarerQol	Self-administered
Cost of intervention	Diaries of time spent on intervention	Self-administered by SLTs, SLTAs and volunteers
Cost of usual care	Diaries of time spent on usual care	SLT collects data from usual treating therapists and participants at baseline, 3, 6, 9 and 12 months
Carer perception of change in communication	Carer COAST	Collected by blinded SLT at baseline. Separate blinded SLT at 6, 9, and 12 months.
Negative effects of treatment	Patient diary to record any difficulties and/or negative impacts of intervention	Patients and carers - central team to send monthly letter reminding to send back in prepaid envelope.
Intervention adherence	Software tailoring checklists. Volunteer and/or assistant feedback forms. Software key files. Puzzle book and telephone support feedback.	Completed by SLT, monitored by central study team. Self-managed practice monitored by central study team. Puzzle book completion and telephone support recorded by member of central study team.

SLT = speech and language therapist; TOMS = Therapy Outcome Measures; COAST = Communication Outcomes After Stroke questionnaire; SLTA speech and language therapy assistant.

### Outcome measures

#### Primary

1. Change in the number of words (personally relevant to the participant) named correctly at 6 months from baseline will be measured by a naming task of 100 pictures presented on the computer.
2. Change in functional communication will be measured by blinded ratings of video-recorded conversations between unfamiliar speech and language therapists blinded to treatment allocation and participants at six months. Conversations will be structured around topics of personal relevance to the participants by the speech and language therapist performing an assessment to ensure the sensitivity of the measure. The same topic guide will be followed by blinded speech and language therapists performing outcome measures. Independent speech and language therapists blinded to treatment allocation and measurement time point will rate the videoed conversations at the project coordinating centre using the activity scale of the Therapy Outcome Measures (TOMS) [26].

#### Key secondary

Improvement in patient perception of communication will be measured using the Communication Outcomes After Stroke (COAST) questionnaire at six months - a patient-centred, patient-reported measure of communication related activity, participation and quality of life [27].

#### Other secondary

Evidence of treatment effect will be measured by repeating all outcome measures at nine and 12 months from baseline, in addition to the primary end point of 6 months. The nine-month time point is included as an interim measure as drop out from the study was found to increase over time in the pilot study [13].

Generalisation of treatment to retrieval of untreated words will be measured using the Naming Objects subtest from the Comprehensive Aphasia Test. Carer perception of communication effectiveness will be measured using the Carer COAST questionnaire [28]. Carer quality of life will be measured using the last five items of the Carer COAST questionnaire [28] and the CarerQol questionnaire [29]. Negative effects of treatment will be reported through diaries. Table 1 summarises the main measures and methods of data collection. Follow-up assessments will be conducted within one month of the target time point.

#### Randomisation, blinding and allocation concealment

Following baseline assessment, the participant will be randomised to one of the three trial intervention arms. Randomisation will be performed by an online randomisation system developed and maintained through the Sheffield Clinical Trials Research Unit (CTRU). The randomisation sequence will be generated in advance by the trial statistician. Randomisation will be stratified by centre (as heterogeneity between centres is expected), and according to severity of word retrieval at baseline, based on percentage scores on the Naming Objects subtest of the Comprehensive Aphasia Test (severe = 10 to 34%, moderate = 35 to 64%, and mild = 65 to 90%). The research speech and language therapist will then inform the participant which group they have been allocated to and draw their attention to the description of this group in the information sheet.

#### Blinding

This is a single blind study. The patient participants are not blind to their treatment allocation. The speech and language therapists performing baseline assessments will do this prior to randomisation. A second speech and language therapist at each site, blinded to group allocation, will perform follow-up assessments. The speech and language therapist setting up the treatment will ask participants not to discuss treatment with the person coming to carry out the follow-up measures. It is possible that unblinding will happen during conversation and the speech and language therapists will be asked to record instances of this. A primary outcome is functional communication in conversation. Video recordings of conversations will be presented in random order to speech and language therapists in the project coordinating centre to rate, blind to treatment allocation and follow-up time. The chief investigator, study manager, statisticians and health economist will all be blind to group allocation.

#### Interventions

##### Usual care control arm

Usual care for this pragmatic study may consist of participation in a range of activities to a greater or lesser extent. Usual care varies across the country in terms of type, frequency and length of provision, and is dependent upon available resources in each locality. Findings from the pilot study confirmed that usual care for four months or more following a stroke may include: face-to-face speech and language therapy targeting language impairment (reading, writing, speaking or understanding); therapy focussing on compensatory communication strategies, provision of communication aids or psychological support; attendance at voluntary support groups or informal communication support from family and friends. Usual care will be recorded retrospectively for three months prior to the study and throughout the study on the case report forms for all groups. Those who are randomised to the usual care group will not receive any project specific intervention.

##### Self-managed computerised therapy intervention

A structured intervention is proposed in addition to usual care as tested in the pilot study. The intervention targets word retrieval as it is one of the challenges most frequently experienced by people with aphasia, restricting their communication. The three components of the intervention are as follows:

- Qualified speech and language therapist assessment, tailoring of exercises and monitoring
  The speech and language therapist at each site will tailor computer exercises to the individual using 100 words of personal relevance chosen by the participant. Photographs imaging the 100 words will be selected from those existing in the software, or can be added from a digital camera or from image stores on a computer. The computer software (StepbyStep© by Steps Consulting Ltd, Gloucester, UK) [30] enables the speech and language therapist to select exercises using these words that follow steps in the therapy process that the therapist would take if delivering it face to face. The speech and language therapist bases the selection of exercises on language skills demonstrated in the initial language assessments. The speech and language therapist will provide initial demonstration of the software exercises, check that the individual is able to use the software and monitor the appropriateness of the tailored exercises. This is expected to take only three to four hours, based on the pilot study [13], as the computer intervention is predominantly self-managed by the patient.
- Regular self-managed practice
  The participant will then be asked to work through the exercises on the computer and be encouraged to practise each day for 20 to 30 minutes. Participants will be given a six-month period to work though the therapy material on the computer and practise using the new vocabulary in their daily lives. As this is a pragmatic trial, those participants who have the software installed on their own computers will not be prevented from continuing to practise if they wish, following the six-month supported intervention time. Any continued use of software beyond the six months will be recorded.
- Volunteer support to assist with treatment adherence and carry over into daily activity
  To enhance treatment adherence, the speech and language therapist will provide training to local volunteers who already have a working relationship with the SLT department or SLT assistants based in the department. They will use the three-hour training programme and instruction book (University of Sheffield, Sheffield, UK) developed and evaluated during the pilot study. The volunteer will be asked to visit the participant once a month for an hour, or every two weeks for half an hour (to suit the patient), carrying out the following tasks: provide technical assistance; observe and encourage use of computer exercises; check results and discuss difficulties; assist the participant to move on to harder tasks in the therapy process pre-programmed by the speech and language therapist; encourage the use of new words in everyday situations, conversation and discussions with family about how to encourage use and encourage re-use of completed exercises over time.
  The participants will be able to contact the volunteer or SLTA by telephone for technical advice on computer use between planned visits if necessary. Volunteers and SLTAs will be asked to complete a feedback form on each face-to-face session with a participant and send it to the therapist to enable them to provide tailored advice and support. They therapists are also encouraged to meet with all the volunteers/SLTAs together every two months for support and discussion of issues arising.
  The majority of the practice time involved in the intervention is self-managed by the participant through regular use of the aphasia computer software. The therapists, SLTAs and volunteers will be asked to keep diaries of resource use showing direct and indirect (telephone and computer set up) time spent and therapist grade (see Table 1).

##### Attention and activity control arm

The third group in this study intends to control for the potential impact of elements of the intervention which of themselves do not provide or require specific speech and language intervention.

Participants randomised to this arm will be provided with generalised activities to carry out and general attention in addition to usual care. On allocation to this group, the speech and language therapists conducting baseline assessments will provide books of standard puzzles that can be purchased from most supermarkets or high street shops. Each book will contain enough activities for one to be carried out each day for at least a month. Examples of puzzles include getting through a maze, spotting the difference between pictures, matching objects that are the same, word searches, Sudoku and so forth. The SLT will provide age appropriate puzzle books that match the participant’s linguistic ability as indicated by the baseline assessments. Puzzle books will be colour coded into levels of easy, medium and hard by the clinicians on the research team centrally and a leaflet will be provided to give speech and language therapists guidance on skills required for each level.

A member of the research team will contact the participants or their carer by telephone or email (whichever is preferred by the participant) once a month to provide the attention similar to that given by volunteers in the intervention arm. They will ask if they are enjoying the activities, how many they have managed to do, whether they would like a new puzzle book sent to them for the coming month and whether they would like the same level of difficulty or an easier or harder one. The participants will also have access to these contact details to enable them to ask for easier or harder books at any time if necessary, again, mimicking the access to the volunteers and SLTAs and type of attention available in the intervention arm.

#### Treatment fidelity

The speech and language therapists delivering this intervention will receive training on how to set up appropriate exercise steps. To enable monitoring of the treatment fidelity, they will be asked to complete a checklist which guides their selection of exercises based on the participant language profile identified during assessment. These will be reviewed centrally by the study quality monitor. Encrypted and anonymised key files from the participants’ software will be returned to the study team to enable comparison of a random selection of exercises provided with the corresponding checklist completed.

As this is a self-managed intervention, adherence of the participants in using the intervention as intended will be monitored. This will be achieved through volunteer or assistant visits with reminders to practise daily and assistance with using the full range of exercises set. The speech and language therapist will also monitor practice through feedback forms provided by the volunteer or assistant. In addition, the software key files returned to the study coordinating team centrally will be reviewed for total practice time and patterns of practice over the six-month treatment period. Practice with the computer for a minimum of 20 minutes three times a week at home on average will be considered per protocol to account for periods of illness and holiday. Volunteer and assistant adherence will be monitored through their feedback forms to the speech and language therapists who will record amounts of support provided. A minimum of four hours in total per patient will be considered per protocol. Adherence to the attention control group will be monitored by recording information regarding the number of puzzles completed and the frequency and duration of telephone support calls received. Usual care will be recorded across the study period for all groups.

#### Data and statistical analysis

##### Sample size

The study aims to recruit 285 participants across 20 speech and language therapy departments (study sites and centres). The sample size of 285 patients in total (95 per arm) is the maximum sample size estimate across the two primary endpoints (word finding ability and functional conversation ability) and key secondary endpoint (patient perception of communication ability) for 90% power and a two-sided significance level of 5%.

##### Assumptions for the sample size calculation

For improvement in word retrieval the estimated effect size is 10%, with a standard deviation (SD) of 17.38%, from an analysis of covariance (based on results of the pilot study [13]). For assessment of conversation the estimated effect size is 0.45 of a SD (with a correlation between baseline and outcome of 0.5 previously observed in the ACT NoW study). For patient-rated improvement using the COAST questionnaire the estimated effect size is 7.2, with a standard deviation (SD) of 13.5 (with an assumed correlation between baseline and outcome of 0.5). The observed dropout rate was 5 out of 33 (15%; 95% CI: 5 to 32%) in the pilot study, which translated to a completion rate of 28/33 (85%; 95% CI: 68 to 95%) [31].

### Internal pilot

The initial phase of the study will be conducted as an internal pilot trial and will include clear criteria to inform decisions about progression. Data from the internal pilot will be included in the final analysis. The internal pilot trial will be limited to six sites (over 25% of the total), representative of the sites which will be in the substantive study. However, during this phase we will recruit and commence set-up processes for all the intended sites. The progression criteria will be reviewed eight months from site set-up of the sixth site in the internal pilot trial. We are estimating that this will be approximately halfway through the recruitment phase, at 22 months from contract start.

The progression will be based on achieving the following criteria: recruitment of no fewer than 30 participants (10% of the target for the full trial), a minimum participant retention rate of 65%, 80% of participants having been offered a volunteer and 70% of participants continuing to be supported by the same volunteer for their six-month treatment period.

### Statistical analysis

Patients with at least one post-randomisation observation will be included in the analysis. Missing data will be described using summary statistics. Data will be checked and cleaned blind to the actual treatment allocation. Data checking will be conducted throughout the study and prior to any analysis of the data.

Primary and key secondary endpoints for the comparisons of control to intervention and active control to intervention will be analysed using a Hochberg testing procedure, which allows for an investigation of all three endpoints whilst maintaining the overall type I error at 5% [32]. This approach has the advantage of not inflating the sample size while maintaining the type I error rate at 5%.

Significance will be declared for the comparison of usual care to intervention if and only if both primary outcomes, word retrieval and conversation, are significant at the 5% level, or if either comparison is significant at 2.5%. If and only if significance is declared for both primary outcomes, a similar comparison of attention control to intervention will be made. Significance will be declared for the comparison of attention control to intervention if and only if both word retrieval and conversation are significant at the 5% level, or if either comparison is significant at 2.5%.

**Figure 2:**
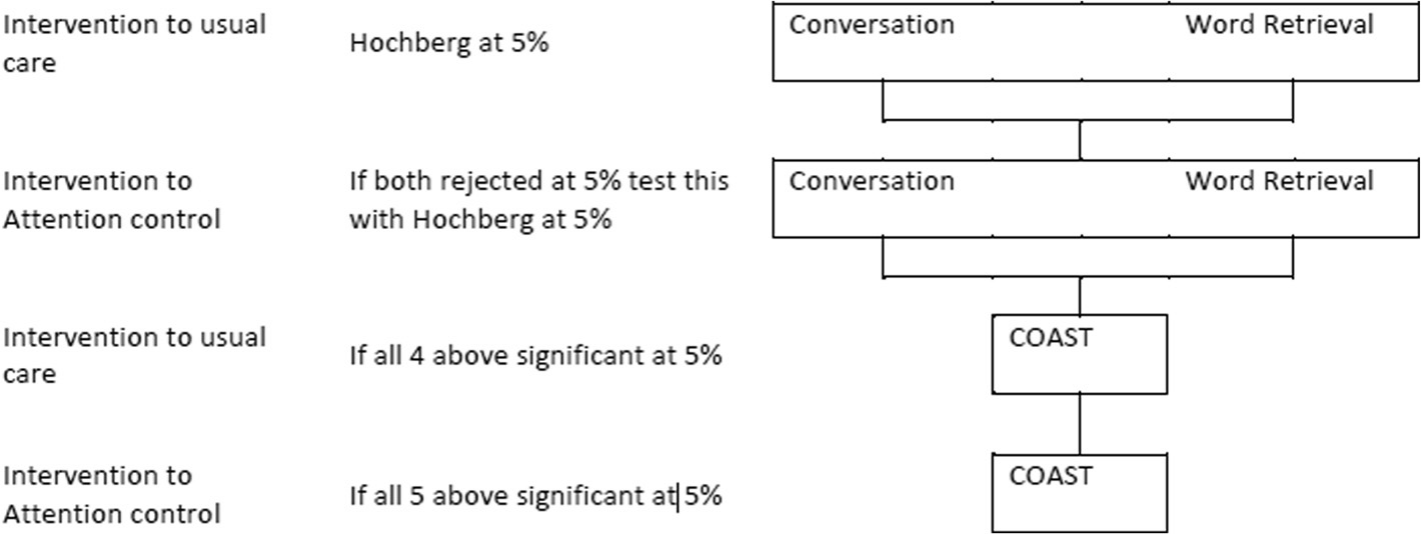
**Statistical testing procedure.** COAST = Communication Outcomes After Stroke questionnaire.

If and only if significance is declared for the comparison of attention control to intervention for both comparisons will the key secondary outcome measure (patient perception of communication and related quality of life, measured using the COAST rating scale) be used in a further comparison of usual care to intervention. If and only if this comparison is significant at the 5% level will the intervention be compared to attention control based on the COAST questionnaire (see Figure 2).

Primary analysis will take an intention-to-treat approach (ITT) for all key measures, and further exploratory analysis of participants who complied with the intervention will be undertaken using the same statistical tests, according to the per protocol principle (PP). Only patients with post-randomisation observations will be included in the primary analysis at six months. A sensitivity analysis responses will be imputed as appropriate with details provided in the statistical analysis plan.

The mean difference in percentage improvement of words named correctly between the treatment and control groups, adjusted for baseline naming ability, will be analysed using an analysis of covariance (ANCOVA). Terms for treatment and baseline will be fitted into the model. Assumptions underlying the analyses will be assessed by inspection of residual plots. Homogeneity of variance will be assessed by plotting the studentised residuals against the predicted values from the model, whilst Normality will be assessed by use of Normal probability plots. If the assumptions for the analysis of variance are violated then appropriate transformations may be applied or alternative analyses may be performed. Similar analyses will be undertaken for the endpoints of functional conversation ability (measured by the activity scale of the Therapy Outcome Measures and patient-reported communication outcomes (the COAST questionnaire). The endpoints at nine and 12 months will be similarly analysed for exploratory purposes. Likewise an investigation of trends over time will be made.

#### Health economic analysis

A cost utility analysis will be undertaken from the NHS and personal social service (PSS) perspective. Due to the use of volunteers to help participants with their use of the computer program we will undertake a supplementary analysis taking a societal perspective. Costs will be estimated for individual patients, including intervention costs and SLT support and coordination time (collected through diaries as shown in Table 1), combined with standard costing sources [33]. In the pilot study we collected other resource use data (on, for example, GP and hospital visits and prescribed medications) via patient and carer diaries, but these did not show important differences between treatment groups and we will not collect such data in the full trial. The EQ-5D questionnaire will be administered at every data collection time point (see Table 1) and will be combined with standard valuation sources to measure the QALYs gained in each treatment arm [34]. An accessible version of the EQ-5D designed for people with aphasia was trialled in the pilot study. This has not been validated but represents a way in which EQ-5D scores can be elicited directly from patients. We will administer this version of the EQ-5D alongside the standard version which will be completed by carers (where the participant has a carer) by proxy. EQ-5D and CarerQoL scores will also be elicited from carers (Table 1).

We developed a Markov model to estimate the cost effectiveness of the computer intervention alongside our previous pilot study. Model parameters were informed by clinical data from the trial. We estimated that the intervention was likely to be cost effective, with an incremental cost effectiveness ratio (ICER) of £3,058 per QALY gained, however results were uncertain and the value of obtaining further (perfect) information was very high (expected value of perfect information (EVPI) was approximately £37 million). This model will be updated with data from the full trial. The third attention control group will be added to the model. Differences between costs and QALYs in the three groups will be described and an incremental analysis will be performed with ICERs calculated. Probabilistic sensitivity analysis will be undertaken to allow the production of cost effectiveness acceptability curves [33] and value of information analyses [35].

### Quality monitoring

The speech and language therapists carrying out outcome measures will receive training on how to deliver the assessments reliably, including scoring criteria and benchmarking of scoring video-recorded assessments with other assessors. Site monitoring visits will also be carried out within the first six months of recruitment (and more frequently if required) to check accurate completion of case report forms, adherence to the protocol for taking consent and discussion of any issues arising.

## Discussion

Chronic aphasia has a considerable impact on a person’s ability to participate in many personal, social and work activities. Despite evidence of the potential for continued language improvement over time with intensive practice of targeted, salient language therapy exercises, opportunities to participate in such ongoing therapy are limited due to resource pressures. There is preliminary evidence of the potential for computer software developed for aphasia therapy to provide new opportunities for ongoing aphasia therapy for patients to participate in for as long as they wish and/or benefit. Indeed, there is an increase in the software options being developed for independent language practice. In order for health services or private practitioners and the public to invest in this option it is crucial to know whether such interventions are clinically and cost effective. This protocol describes the first fully powered RCT of long term aphasia therapy delivered through self-managed computerised language exercises, with the support of volunteers or assistants. The protocol also describes the process for the first full economic analysis of a computerised aphasia intervention.

The therapy under investigation is a complex intervention of word finding therapy for aphasia. Previous studies, including the pilot study informing this protocol, have shown that people with aphasia learn the words that are meaningful to them more successfully than those they have little functional need for. As in many rehabilitation interventions, this therapy is driven by the patient’s own goals. Communication needs to achieve different goals are discussed with the therapist and a range of words key to achieving those goals are identified. The personalisation of the therapy goals requires some personalisation in the measurement of whether they have been achieved. The design of outcome measures which can be used to make group comparisons whilst maintaining accurate measurement of individual goals was a challenge. Discussions resulted in two primary outcome measures. The first measures word finding ability whereby all participants have comparable word finding scores from naming 100 pictures. Measurement of word finding of personally meaningful and useful words is achieved by allowing the 100 words to be different for each individual according to their goals. Similarly, the second measures ability to communicate in conversation. All conversations are rated using the activity scale of the TOMS so group comparisons can be made. Measurement of individual goals is maintained by structuring the conversations around the communication areas identified as personally relevant by each individual.

Other key issues in designing the protocol arose from the pragmatic nature of the study. First of all, in order to recruit sufficient numbers of participants, at least 20 speech and language departments are required across the United Kingdom. This will allow generalisation of the results to the population of the United Kingdom. However, we expect considerable variation in the usual care provided to people with aphasia in the long term post stroke, with some sites providing impairment based interventions face to face for several months, and others limiting their service provision to hospital care or for a few weeks post-hospital discharge only. The intervention being tested could be implemented in addition to any existing services in practice. The study therefore uses an adjunct design whereby patients continue to receive any ongoing care. This may have the added benefit of recruiting those patients who would not wish to be randomised away from usual care. To account for the heterogeneity expected, the randomisation is to be stratified by site, ensuring representation of different models of usual care in each study group.

There are challenges in involving people with aphasia in research, as they often have difficulty reading or understanding spoken language. The protocol for obtaining informed consent therefore needed careful consideration. In order to adhere to the Mental Capacity Act (2005) in England and the Adults with Incapacity Act (2000) in Scotland, a consent support tool has been included to identify reading and spoken comprehension ability of individuals and recommend the style of information that is likely to best inform each individual. The tool also identifies those participants whose aphasia severity is such that they are unlikely to understand the information sufficiently to provide informed consent and therefore need the involvement of a carer, relative or legal representative. In addition, the reading deficits make completion of standard questionnaire tools such as the EQ-5D difficult and unreliable. We therefore developed and tested an accessible EQ-5D which uses illustrations and visually represented extent of difficulty.

There are three groups in the study. Introducing a three-way comparison increases the required sample size and therefore poses a potential threat to the power of the study if recruitment is insufficient. However, a three-way comparison is not required here as the first question to answer is whether the computerised intervention is more effective than usual care alone. Only if the answer to the first question is positive is there a need to ask whether it is the speech and language therapy components that cause the effectiveness shown. In this case a second comparison of computer intervention to activity and/or attention control needs to be carried out. The Hochberg analysis was chosen to allow for these consecutive comparisons, thus also maintaining a more achievable sample size.

Finally, the set-up of 20 individual speech and language therapy departments to participate in the study is a considerable challenge, in which service support and excess treatment costs must be negotiated at each site independently, with a large range of local policies and financial restrictions across the United Kingdom.

## Trial status

The study has received ethical approval and fifteen participants have been recruited from the first five sites set up.

## Abbreviations

ANCOVA: Analysis of covariance
CAT: CIAT, Constraint induced aphasia therapy
COAST: Communication Outcomes After Stroke
CST: Consent support tool
CTRU: Clinical Trials Research Unit
EVPI: Estimated value of perfect information
ICER: Incremental cost effectiveness ratio
ISRCTN: International standard randomised controlled trial number
MOAT: Model oriented aphasia therapy
NHS: National Health Service
NIHR: National Institute of Health Research
PSS: Personal social service
QALYs: Quality-adjusted life years
RCT: Randomised control trial
SLT: speech and language therapy
SLTA: Speech and language therapy assistant
TOM: Therapy Outcome Measures

## References

[1] Department of Health (2007). National Stroke Strategy.

[2] Brady MC, Kelly H, Godwin J, Enderby P (2012). Speech and language therapy for aphasia following stroke. Cochrane Database Syst Rev.

[3] Laska A, Kahan T, Helblom A, Murray V, Von Arbin MA (2011). Randomized controlled trial on very early speech and language therapy in acute stroke patients with aphasia. Cerebrovasc Dis Extra.

[4] Bowen A, Hesketh A, Patchick E, Young A, Davies L, Vail A, Long A, Watkins C, Wilkinson M, Pearl G, Lambon Ralph M, Tyrrell P (2012). Clinical effectiveness, cost effectiveness and service users’ perceptions of early, well-resourced communication therapy following a stroke, a randomised controlled trial (The ACT NoW Study). Health Technol Assess.

[5] Meinzer M, Djundja D, Barthel G, Elbert T, Rockstroh B (2005). Long term stability of improved language functions in chronic aphasia after constraint-induced aphasia therapy. Stroke.

[6] Raymer A, Beeson P, Holland A, Kendall D, Maher L, Martin N, Murray L, Rose M, Thompson CK, Turkstra L, Altmann L, Boyle M, Conway T, Hula W, Kearns K, Rapp B, Simmons-Mackie N, Gonzalez Rothi LJ (2008). Translational research in aphasia: from neuroscience to neurorehabilitation. J Speech Lang Hear Res.

[7] Kurland J, Baldwin K, Tauer C (2010). Treatment-induced neuroplasticity following intensive naming therapy in a case of chronic Wernicke’s aphasia. Aphasiology.

[8] Pulvermuller F, Berthier M (2008). Aphasia therapy on a neuroscience basis. Aphasiology.

[9] Pulvermuller F, Neininger B, Elbert T, Mohr B, Rockstroh B, Koebbel P, Taub E (2001). Constraint induced therapy of chronic aphasia after stroke. Stroke.

[10] Cherney L, Patterson J, Raymer A, Frymark T (2008). Evidence-based systematic review: effects of intensity of treatment and constraint-induced language therapy for individuals with stroke-induced aphasia. J Speech Lang Hear Res.

[11] Barthel G, Meinzer M, Djundja D, Rockstroh B (2008). Intensive language therapy in chronic aphasia: which aspects contribute most?. Aphasiology.

[12] David R, Enderby P, Bainton D (1982). Treatment of acquired aphasia: speech therapists and volunteers compared. J Neurol Neurosurg Psychiatr.

[13] Palmer R, Enderby P, Cooper C, Latimer N, Julious S, Paterson G, Dimairo M, Dixon S, Mortley J, Hilton R, Delaney A, Hughes H (2012). Computer therapy compared with usual care for people with long standing aphasia post stroke: a pilot randomized controlled trial. Stroke.

[14] Cherney LR (2010). Oral Reading for Language in Aphasia (ORLA): evaluating the efficacy of computer-delivered therapy in chronic nonfluent aphasia. Top Stroke Rehabil.

[15] van de Sandt-Koenderman M (2011). Aphasia rehabilitation and the role of computer technology: can we keep up with modern times?. Int J Speech Lang Pathol.

[16] Fink R, Breecher A, Schwarz M, Robey R (2002). A computer-implemented protocol for treatment of naming disorders: evaluation of clinician-guided and partially self-guided instruction. Aphasiology.

[17] Mortley J, Wade J, Enderby P (2004). Superhighway to promoting a client-therapist partnership: using the internet to deliver word-retrieval computer therapy monitored remotely with minimal speech and language therapy input. Aphasiology.

[18] Department of Health (2006). Our Health, Our Care, Our Say: a New Direction for Community Services.

[19] Katz R, Wertz R (1997). The efficacy of computer-provided reading treatment for chronic aphasic adults. J Speech Lang Hear Res.

[20] Palmer R, Mortley J. How I offer impairment therapy (2): from idealism to realism, step by step. Speech & Language Therapy in Practice. 2011; Winter:29–32.

[21] Palmer R, Enderby P, Paterson G (2013). Using computers to enable self-management of aphasia therapy exercises for word finding: the patient and carer perspective. Int J of Language and Communication Dis.

[22] Latimer NR, Dixon S, Palmer R (2013). Cost-utility of self-managed computer therapy for people with aphasia. Int J Technol Assess Health Care.

[23] Boutron I, Moher D, Altman D, Schulz K, Ravaud P, for the Consort group (2008). Extending the CONSORT statement to randomized trials of nonpharmacologic treatment: explanation and elaboration. Ann Intern Med.

[24] Swinburn K, Porter G, Howard D (2004). Comprehensive Aphasia Test.

[25] Jayes M, Palmer R (2013). Initial evaluation of the consent support tool: a structured procedure to facilitate the inclusion and engagement of people with aphasia in the informed consent process. Int J Speech Lang Pathol.

[26] Enderby PM, John A, Petheram B (2006). Therapy Outcome Measures for Rehabilitation Professionals.

[27] Long A, Hesketh A, Paszek G, Booth M, Bowen A (2008). Development of a reliable self-report outcome measure for pragmatic trials of communication therapy following stroke: the Communication Outcome after Stroke (COAST) scale. Clin Rehabil.

[28] Long AF, Hesketh A, Bowen A, on behalf of the ACT NoW Study (2009). Communication outcome after stroke: a new measure of the carer's perspective. Clin Rehabil.

[29] Brouwer W, van Exel N, van Gorp B, Redekop W (2006). The CarerQol instrument: a new instrument to measure care-related quality of life of informal caregivers for use in economic evaluations. Qual Life Res.

[30] Steps Consultancy Ltd: StepbyStep© software. [http://www.aphasia-software.com]

[31] Julious SA (2009). Sample Sizes for Clinical Trials.

[32] Hochberg Y, Tamhane AC (1987). Multiple Comparison Procedures.

[33] Curtis L. Unit Costs of Health and Social Care. University of Kent at Canterbury: Personal and Social Services Research Unit; 2011.

[34] Dolan P (1997). Modeling valuations for EuroQol health states. Med Care.

[35] Griffin S, Welton NJ, Claxton K (2010). Exploring the research decision space: the expected value of information for sequential research designs. Med Decis Making.

